# Art therapy to reduce burnout and mental distress in healthcare professionals in acute hospitals: a randomised controlled trial

**DOI:** 10.1136/bmjph-2024-002251

**Published:** 2025-08-03

**Authors:** Megan Tjasink, Catherine Elizabeth Carr, Paul Bassett, Gehan Soosaipillai, Dennis Ougrin, Stefan Priebe

**Affiliations:** 1 Unit for Social and Community Psychiatry WHO Collaborating Centre for Mental Health Services Development Centre for Psychiatry and Mental Health Wolfson Institute of Population Health, Queen Mary University of London, London, UK; 2 Barts Health NHS Trust, London, UK; 3 East London NHS Foundation Trust, London, UK; 4 Statsconsultancy Ltd, Amersham, UK; 5 Guy's and St Thomas’ Hospitals NHS Trust, London, UK; 6 Youth Resilience Unit, Centre for Psychiatry and Mental Health, Wolfson Institute of Population Health, Queen Mary University of London, London, UK; 7 Centre for Psychosocial Medicine, University of Hamburg, Hamburg, Germany

**Keywords:** Occupational Medicine, Anxiety, Health Personnel, Preventive Psychiatry, Depression

## Abstract

**Introduction:**

Burnout and mental distress are prevalent among healthcare professionals (HCPs), particularly in acute hospital settings. This study evaluated the effectiveness of a structured group art therapy intervention in reducing burnout and associated mental distress in HCPs.

**Methods:**

We conducted a multicentre, unblinded, randomised, parallel assignment, waitlist-controlled trial in four National Health Service secondary care hospitals in London, UK, between 4 May 2023 and 5 March 2024. A total of 129 HCPs with moderate-to-severe risk of burnout or levels of perceived stress were randomly assigned to either group art therapy (6 weekly 90-min sessions) or a waitlist control.

The primary outcome was change in emotional exhaustion as a core dimension of burnout, measured using the Maslach Burnout Inventory-Human Services Survey. Secondary outcomes were the other two burnout dimensions: depersonalisation and personal accomplishment, as well as perceived stress, measured on the perceived stress scale, anxiety, assessed on the generalised anxiety disorder seven-item scale, and depression, measured on the eight-item patient health questionnaire depression scale. Outcomes were assessed at baseline and 6 weeks postintervention/control period. Intervention group outcomes were also assessed at 3-month follow-up.

**Results:**

Primary outcome data were obtained from 115 (89%) of 129 participants, who represented a range of clinical specialties and professions. Emotional exhaustion scores were significantly lower in the intervention group compared with the control group at 6 weeks (adjusted mean difference: 4.8; 95% CI 2.4 to 7.3; p<0.001). Significantly more favourable scores were also found in depersonalisation, perceived stress, anxiety and depression in the intervention group. Gains were sustained at 3-month follow-up.

**Conclusions:**

Six weekly sessions of group art therapy can significantly reduce burnout risk and mental distress in HCPs from different professional backgrounds in acute hospital settings. Wider implementation of the intervention should be considered.

**Trial registration number:**

ClinicalTrials.gov ID: NCT05728086.

**WHAT IS ALREADY KNOWN ON THIS TOPIC**

- Burnout is a major concern for healthcare professionals (HCPs) and healthcare systems. Most interventions show limited impact. Pilot studies suggested that group art therapy could be effective.

**WHAT THIS STUDY ADDS**

- This first full randomised controlled trial shows that six sessions of specified group art therapy significantly reduce burnout and mental distress in hospital-based HCPs.

**HOW THIS STUDY MIGHT AFFECT RESEARCH, PRACTICE OR POLICY**

- Findings support its use in staff support services and further research into scalability and long-term outcomes.

## Introduction

 Burnout has long been recognised as a serious problem affecting healthcare professionals (HCPs).^1^ Following the COVID-19 pandemic, the levels of HCP burnout appear to have substantially risen worldwide.^2 3^ This has led to concerns that burnout could jeopardise the global provision of quality healthcare.^4^ Caused by exposure to ongoing work-related stressors,^5^ such as overly burdensome workloads and under-resourcing,^6^ burnout is an occupational phenomenon^7^ increasingly prevalent even in HCPs who are newly qualified or still in training.^8^ According to the model by Maslach and Jackson, burnout is characterised by three dimensions, that is, emotional exhaustion, depersonalisation and a lack of personal accomplishment.^9^ Emotional exhaustion is considered the key dimension of the three.^10^ Meta-analyses have found that the burnout of HCPs can lead to poor clinical outcomes, reduced patient safety and lower patient satisfaction.^11^ It affects staff retention, undermines sustainable healthcare organisation^12^ and places a large financial burden on often already overstretched healthcare systems.^13^ Moreover, it can negatively impact HCPs’ personal lives^14^ and is strongly associated with mental distress, such as depression and suicidality.^15 16^ While research in burnout and psychosocial distress in HCPs has burgeoned over the past decade,^17 18^ there is no consensus on effective interventions. Systematic reviews of interventions show inconsistent findings^19^ with small or negligible effect sizes^20^,^22^ and conclude that there is a pressing need for more research, particularly high-quality randomised controlled trials (RCTs) testing a wider breadth of tailored interventions.^20 21^

A systematic review of heterogeneous studies assessing art therapy-based interventions for HCPs^23^ found that such interventions are promising as an effective treatment. However, results were not suitable for a meta-analysis, warranting further investigation. Against this background, the present RCT tests an art therapy-based approach developed for HCPs in acute hospitals that builds on a previous pilot trial^24^ with medical doctors in oncology and palliative care. The RCT tests the effectiveness of six weekly sessions of specified group art therapy for HCPs from different specialties and professional backgrounds.

## Materials and methods

### Study design and setting

In this parallel assignment randomised waitlist-controlled trial, HCPs were screened for heightened risk of burnout and perceived stress. They were then randomised to group art therapy or a waiting group with no specific treatment. The allocation ratio was 1.35:1 (intervention: control) to account for potential cluster effects in the intervention group. Those randomised to the waitlist control arm were offered group art therapy after their waitlist period was complete. Participants were recruited from four major hospitals in London from a large National Health Service (NHS) Trust serving more than 2.5 million patients and employing around 18 000 staff. The hospitals provide specialist and emergency services and encompass a teaching hospital and large cancer and cardiovascular centres.

The first, second and last authors, representing the host NHS Trust and affiliated academic partners, designed the trial with the input of an independent statistician. The trial was managed within the hosting NHS Trust. A Trial Steering Committee and Trial Management Committee served data monitoring purposes. The trial protocol was registered on ClinicalTrials.gov on 10 February 2023. ID: NCT05728086.

### Participants and procedures

Potential participants received information about the study through hospital communication channels, such as departmental emails, meetings and circulars. HCPs who contacted the research team with an expression of interest were followed up with further information. Eligibility criteria were moderate to severe risk of burnout scored on one or more of the Maslach Burnout Inventory-Human Services Survey (MBI-HSS) scales: emotional exhaustion score of ≥17 (scale of 0–54), or depersonalisation score of ≥7 (scale of 0–30), or personal accomplishment score of ≤38 (scale of 0–48) and/or perceived stress assessed on the perceived stress scale (PSS10) (perceived stress of ≥14). Exclusion criteria were diagnosis of or treatment for a serious depressive disorder, a personality disorder or psychosis and attempted suicide or plans to commit suicide within the past 12 months. Hospital employees not providing direct patient care were excluded. Written informed consent was obtained by a member of the research team via email or in-person. Participants consenting via email were given the option to discuss eligibility and consent with the research team in-person or on the phone. A participant was considered to have completed the study if they submitted the end-of-treatment (6 week) outcome measures. There was no minimum session attendance required for inclusion in the intention-to-treat (ITT) analysis. However, session attendance was recorded, and the median number of sessions attended is reported in the Results section. Study procedures are summarised according to the Standard Protocol Items: Recommendations for Interventional Trials 2013 Statement^25^ in online supplemental file 1: study procedures.

### Intervention

The intervention tested is a specified short course of group art therapy developed for HCPs in acute hospitals, delivered by qualified art therapists. Art therapy is a regulated psychological profession that uses non-verbal visual art processes as a key therapeutic element. It combines psychological theories and techniques with creative processes,^26^ providing a holistic approach to health and well-being through biopsychosocial theoretical frameworks.^27^ Summarised according to the Template for Intervention Description and Replication (TIDieR) checklist^28^ (online supplemental file 2: TiDier table), the intervention was adapted from an art therapy programme designed for oncology and palliative care doctors.^24^ Core elements of the original intervention, such as dose and delivery (6 weekly 90 min sessions delivered in groups by a professional art therapist), remain as does the dual focus on individual and social factors through a combination of individual and collaborative art making and reflection. Adaptations were made to increase relevance to a broader range of HCPs and to integrate trauma-informed art therapy methods developed for staff support during the COVID-19 pandemic.^29^ These adaptations include more focus on experimental and exploratory art-making techniques and the removal of sessions specifically focused on dealing with death and dying designed specifically for HCPs working in oncology and palliative care. Sessions are structured but not inflexible. Each session has a different focus and set of aims, but all sessions follow a similar format comprising: (1) socialisation; (2) introduction to the workshop theme, art materials and processes; (3) art-based warm up or grounding exercise; (4) individual and/or collaborative art making; (5) sharing and viewing art products and processes and (6) reflective group discussion. Examples of artwork created by HCPs in session 2: ‘exploring natural objects and experimental art making’, session 4: ‘transforming images (collaborative problem solving)’ and session 5: ‘create, destroy and transform’ are included in online supplemental file 3: example artwork. An expert focus group (NHS art therapists working with staff in medical settings) and a Patient and Public Involvement and Engagement (PPIE) panel of experts by experience were consulted as a part of the intervention development process, which was informed by the UK Medical Research Council and the National Institute for Health and Care Research^30^ new framework for developing and evaluating complex interventions.^31^

Stakeholder consultation and exploratory testing found late afternoon/early evening to be the preferred time of day for sessions, with later evening for a subgroup of operating theatres staff, such as anaesthetists. This was because the prospect of taking time to attend during scheduled working hours was generally anticipated to add unwelcome pressure, posing challenges to team working and patient care. Sessions took place in non-clinical spaces, such as seminar or meeting rooms within participating hospitals.

Strategies to assess and improve adherence to the intervention were implemented. Art therapists delivering the intervention received additional training in the approach and clinical supervision from an experienced art therapist. Adherence to the manual was assessed using a scale consisting of 14 questions and a five-point confidence scale (see online supplemental file 4: therapist self-adherence form), which was adapted from a large trial comparing different arts therapies with talking therapy for people with different mental illnesses.^32^ Questions were based on a framework of three categories. (1) The content of the intervention—adherence to exercises, topics or components as laid out in the intervention manual. (2) The techniques employed in the intervention—how it was delivered, for example, group facilitation skills and normalisation of a range of emotions. (3) The degree of individualisation of the intervention. Adherence was self-recorded by therapists after each session and by an independent observer, who attended and rated a purposive sample of sessions, selected to include a spread of therapists and hospital sites.

### Comparator

A waitlist control group was used to compare the outcomes of those receiving the intervention with those who received no specific treatment during the trial. The participating hospitals provided staff well-being programmes and a specialist staff support psychology service, but data regarding potential engagement of control group members with existing individual or organisational support were not collected. There was no restriction on concomitant care for the intervention or waitlist control groups during the trial.

### Outcomes

The primary outcome was emotional exhaustion as measured on the MBI-HSS, a widely established instrument for assessing the three dimensions of burnout in professions like healthcare, social work and education.^33^Maslach and Jackson’s foundational paper, ‘The measurement of experienced burnout’, introduces and validates the instrument.^9^ The Maslach Burnout Inventory manual directs that its three scales—emotional exhaustion, depersonalisation and personal accomplishment—should not be combined into a single score as the scales were developed to capture different dimensions of burnout. Maslach *et al* consider emotional exhaustion to be the central dimension of burnout, which often precedes and predicts the other two dimensions.^10 34^

Secondary outcomes were the other two burnout dimensions: depersonalisation and personal accomplishment (MBI-HSS), as well as perceived stress using the PSS10^35^; levels of anxiety, assessed on the generalised anxiety disorder seven-item scale (GAD-7)^36^ and depression, measured on the eight-item patient health questionnaire depression scale (PHQ-8).^37^ While the MBI-HSS and PSS10 measure occupational phenomena, the GAD-7 and the PHQ-8 are the mental health outcomes for psychiatric disorders commonly associated with burnout.^38^ Outcomes were assessed at baseline and at the end of the intervention or waiting period. In the intervention group, outcomes were also assessed 3 months after the end of art therapy to explore whether changes during the intervention were sustained. Additionally, participant experiences were collected from both groups after receiving art therapy using a feedback questionnaire comprised of one Likert scale question about helpfulness of the intervention (range 1–5 from ‘very unhelpful’ to ‘very helpful’) and seven further nominal questions.

Adverse events and reactions were documented.

### Sample size

As there is no established clinically significant effect size for reducing burnout, we designed the study to detect an effect size of 0.6 on a 5% significance level and with 80% power. This required 90 participants (45 per arm) before adjustment for design effect. Adjusting for potential cluster effects in the intervention group, based on the cluster size of 8 (number of participants per group) and an intracluster correlation of 0.05, the amount that the sample size needed to be inflated to account for design effect was 1.35. Applying this inflation factor to a sample of 45 in the intervention group brought the number to 61. Allowing for 15% attrition, this indicated 52 in the control group and 70 in the intervention group, giving a total sample size of 125 (53+72).

### Randomisation and masking

Following self-enrolment on the completion of online baseline questionnaires (to ensure allocation concealment), participants were allocated to waitlist control or intervention conditions using a computer-generated blocked randomisation list with permuted block sizes. An online provider (sealed envelope) randomised the first 80 cases, and the study statistician (PB) provided randomisation for remaining participants. Once randomised, HCPs were allocated to an art therapy group by the trial manager. Where possible, options were offered to accommodate clinical and personal commitments. Due to the nature of the intervention, neither participants nor those delivering it were able to be masked to treatment group assignment. Follow-up outcomes were rated by all participants in the intervention and control group online with no presence of a researcher or therapist.

### Patient and public involvement

The original 6-week art therapy course, which formed the basis of the intervention tested in this trial, was coproduced with a group of NHS doctors to tailor it to their working context.^24^ A PPIE panel comprised of experts by experience (HCPs from several professional groups and with the personal experience of art therapy for occupational stress) was involved at several stages of the trial. The panel contributed to the development and assessment of trial materials. They checked all participant information and publicity material for tone and ease of engagement. Panel members tested the online database, used to self-report outcomes, for ease of use and burden of time. The interface was adapted to be more user-friendly following their feedback. The panel also suggested an adjusted scale and additional question for the participant feedback questionnaire, and both were adopted. They were consulted on the intervention manual and about recruitment strategy, contributing to the identification of high need or ‘forgotten’ professional groups, such as operating theatre practitioners and pharmacists. The PPIE panel was represented on the Trial Steering Committee and a panel member has contributed here as a coauthor. The collaborative approach adopted in planning and delivery of this trial was likely to have been a key factor in the positive response from participants and all hospitals taking part.

### Statistical methods

Recruitment and missing data between the two groups were recorded in a Consolidated Standards of Reporting Trials (CONSORT)^39^ diagram. All analyses were performed on an ITT basis, as prespecified in the study protocol. Stata (version 15.1) was used for analysis.

### Comparison of intervention and waitlist controls

The first analyses descriptively show the primary and secondary outcomes at baseline. Subsequent analyses compared the two groups at postintervention (6 weeks). Since art therapy was delivered in groups, the analysis was performed using linear mixed (multilevel) models, specifying an independent covariance structure. The therapy group to which the participant was assigned was included as a random effect in the models. Subjects in the control group were assumed to be in their own cluster. In the analysis, the postintervention measurement was used as the outcome variable, with the equivalent outcome at baseline included as a covariate (fixed factor).

For the outcomes drawn from the MBI-HSS and PSS10 outcomes, all individual items were completed, allowing the scores to be calculated for all participants starting the questionnaire. For the PHQ-8 and GAD-7 outcomes, not all items were completed for 14 (10.8%) of subjects. Where only one item from the questionnaire was not completed, a value was assumed equal to the mean of the completed items, enabling the score for these subjects to be calculated. Where two or more items were missing, the scores were considered as missing. All statistical tests were two sided, and a p value of <0.05 was considered statistically significant.

### Methods for additional analyses

We explored whether any improvements were sustained during the 3-month follow-up period. For this, analysis of change from postintervention to follow-up time point was performed for the intervention group using linear mixed (multilevel) models to account for group delivery. The outcome variable was the change in outcome between timepoints. The cluster to which the participant was assigned was included as a random effect in the models, while no fixed effects were included.

### Missing data and attrition

Statistical analysis was performed as an ITT analysis. Missing data have been reported.

### Role of the funding source

The funder had no role in the study design, data collection, data analysis, data interpretation or writing of the report.

## Results

Between 10 March 2023 and 27 December 2023, we randomly assigned 53 participants to the waitlist control and 76 participants to art therapy. Only two interested HCPs screened out postbaseline due to the low risk of burnout (MBI-HSS) and low stress (PSS10). 14 participants (11%) did not complete the postintervention assessment: 6 from the intervention group and 8 from the control group. The overall attrition rate was within the range predicted in the study protocol. Missing data were minimal and not imputed; only complete case data were used in the primary outcome analysis.

**Figure 1:**
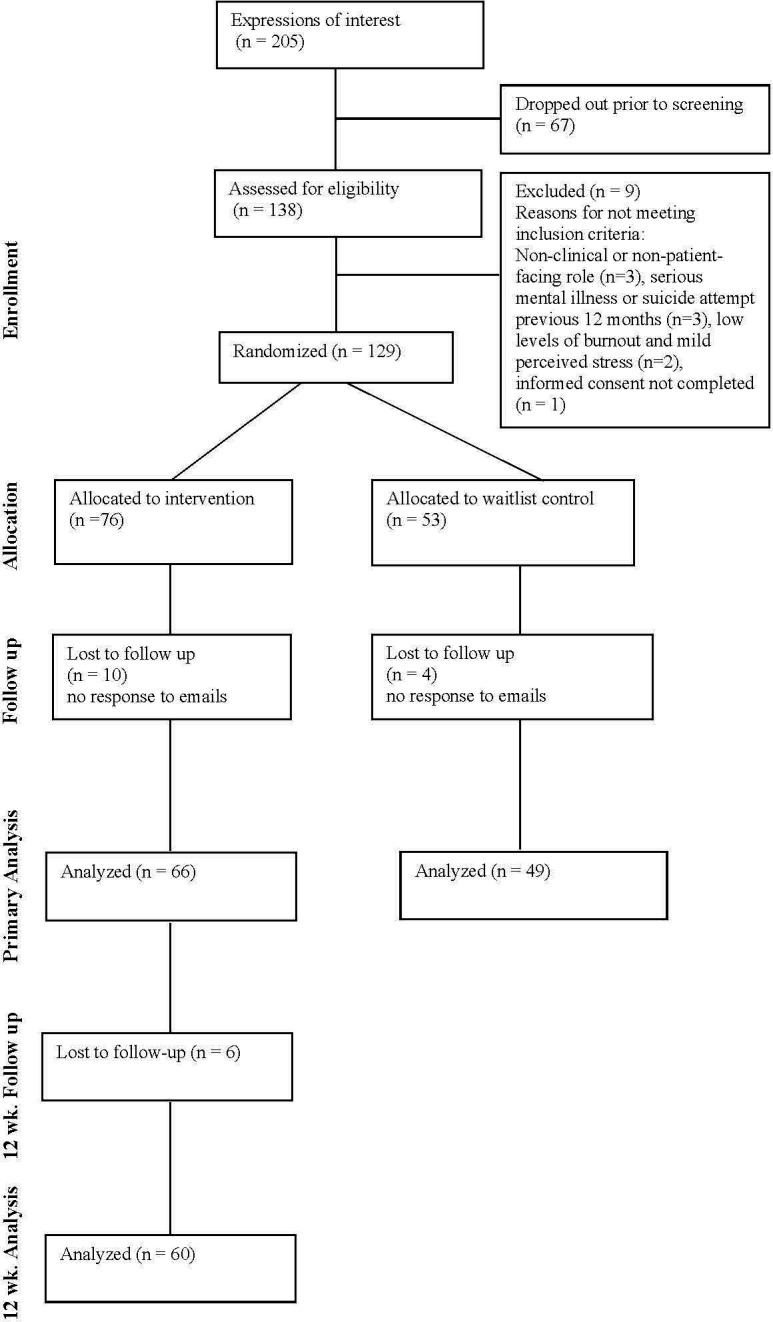
CONSORT^39^ diagram showing the flow of participants through the trial. CONSORT, Consolidated Standards of Reporting Trials.

The flow of participants through the trial is summarised in a CONSORT^40^ diagram (figure 1).

### Baseline data

Participant characteristics: participants’ ages ranged from 20 to 67 years (*M*=36.5, SD=10.3) with 86% identified as female and 16% did not speak English as a first language. The ethnicity of participants varied as is common in London’s HCP workforce.^41^ HCPs from different professional backgrounds took part. The largest professional subgroup was of doctors (n=54). Participants were drawn from various clinical areas, with highest numbers from oncology and emergency departments. Participant characteristics are outlined in table 1. Context-specific reporting of ethnic group classifications to capture the diversity of the population has been used in accordance with NIHR *INCLUDE Ethnicity Framework: improving the inclusion of underserved groups in clinical research.*^30^

**Table 1:** Participant demographic characteristics and dependent variables at baseline

Age	Control (n=53)	Intervention (n=76)
Range (min–max)	22–63	20–67
Mean	36.5 (SD 9.7)	36.3 (SD 10.7)
Sex	n (%)	n (%)
Women	43 (81)	68 (91)
Men	10 (19)	7 (9)
Did not answer		1
Ethnicity	n (%)	n (%)
White—English, Welsh, Scottish, Northern Irish or British	21 (39.6)	30 (39.5)
White—Irish	1 (1.9)	5 (6.6)
Any other White background	7 (13.2)	8 (10.5)
Asian/Asian British—Indian	10 (18.7)	6 (7.9)
Asian/Asian British—Pakistani	1 (1.9)	2 (2.6)
Asian/Asian British—Chinese	1 (1.9)	5 (6.6)
Any other Asian background	4 (7.5)	8 (10.5)
Black/Black British—African	4 (7.5)	4 (5.3)
Any other Black/Black British background	0 (0.0)	1 (1.3)
Mixed. White and Asian	1 (1.9)	4 (5.3)
Mixed. White and Black	1 (1.9)	1 (1.3)
Any other mixed or multiple ethnic background	2 (3.8)	2 (2.6)
Language	n (%)	n (%)
English as first language	40 (75.5)	58 (76.3)
Bilingual with English as 'one of' first languages	4 (7.5)	5 (6.6)
English not first language	9 (17.0)	13 (17.1)
Job role	n (%)	n (%)
Junior Doctor	14 (26.4)	20 (26.3)
Allied Health Professional	9 (17.0)	13 (17.1)
Consultant Doctor	12 (22.6)	8 (10.5)
Nurse	7 (13.2)	13 (17.1)
Clinical Nurse Specialist	4 (7.5)	8 (10.5)
Pharmacy	4 (7.5)	6 (7.9)
Psychological Specialist	2 (3.8)	4 (5.3)
Healthcare Assistant	0 (0.0)	2 (2.6)
Midwife	1 (1.9)	2 (2.6)
Clinical specialty		
Oncology	8 (15.0)	16 (21.1)
Emergency	8 (15.0)	10 (13.2)
Surgery	3 (5.7)	8 (10.5)
Cardiac	4 (7.5)	7 (9.2)
Ophthalmology	2 (3.8)	5 (6.6)
Anaesthetics	3 (5.7)	4 (5.3)
Renal	3 (5.7)	3 (3.9)
ICU	3 (5.7)	3 (3.9)
Paediatrics	3 (5.7)	2 (2.6)
Palliative care	2 (3.8)	2 (2.6)
Other	14 (26.4)	16 (21.1)

Outcome	Control		Intervention	
	N	Mean±SD	N	Mean±SD
Primary outcome				
MBI-HSS emotional exhaustion	53	30.0±9.6	76	31.5±10.3
Secondary outcomes				
MBI-HSS depersonalisation	53	7.6±4.9	76	9.9±6.5
MBI-HSS personal accomplishment	53	34.3±6.2	76	33.7±6.5
PSS10	53	22.1±5.4	76	22.2±6.5
PHQ-8	53	8.1±4.0	75	7.9±5.3
GAD-7	53	6.8±4.1	74	7.5±4.9

GAD-7, generalised anxiety disorder seven-item scale; HCA, healthcare assistant; ICU, intensive care unit; MBI-HSS, Maslach Burnout Inventory-Human Services Survey; PHQ-8, eight-item patient health questionnaire depression scale; PSS10, perceived stress scale.

The baseline average scores for emotional exhaustion put participants in both groups within the high-risk range (score>27) for burnout. Average baseline scores for perceived stress, depersonalisation and personal accomplishment were all within the range reflecting moderate severity according to the scale definitions, while anxiety and depression were within the mild range.

### Intervention delivery

Four Health and Care Professions Council registered art therapists with the experience of working in NHS settings delivered the sessions according to specifications of the manual. A total of 108 sessions (18 art therapy groups receiving 6 sessions each) were delivered across four inner city London hospitals (St Bartholomew’s Hospital, The Royal London Hospital, Whipps Cross Hospital and Newham University Hospital) between 4 May 2023 and 5 March 2024. Each session was conducted by a single therapist. Group sizes ranged from 5 to 11 participants and the median number per group was 8 (IQR 5–9). Most groups (n=15) were delivered in the early evening between 17:30 and 19:30, one group ran from 11:15 to 12:45 and there were two later evening groups ending at 21:00. Participants attended a median of four out of six sessions (IQR 3–5). Six participants did not attend any sessions: one left the hospital, one changed their mind, for two, timing was incompatible with their workplan and two changed their work schedule after enrolling. More than 1200 artworks were created by participants in the sessions.

### Adherence

Adherence to the intervention manual was high with 98.7% of adherence criteria met according to therapist ratings. Observers assessed adherence at 92.8%. Both scored a 91.7% confidence rating using a five-point confidence scale.

### Outcomes

Primary outcome data at the end of the intervention or waiting period were collected from 115 (89%) of 129 participants. The results are summarised in table 2.

**Table 2:** Baseline and postintervention (6 weeks) outcome data in the intervention and control group; the group differences at the end of the intervention or waiting period are adjusted for the baseline score of the given scale and in the intervention group also for cluster effects

Outcome	Group	N	Baseline Mean±SD	Postintervention Mean±SD	Adjusted difference Mean (95% CI)	P value	Cohen’s d (95% CI)
Primary outcome							
MBI emotional exhaustion	Control	49	30.0±9.7	31.1±10.6			
	Intervention	66	31.7±10.4	27.5±9.8	−4.8 (−7.3 to −2.4)	**<0.001**	−0.48 (−0.73 to −0.24)
Secondary outcomes							
MBI depersonalisation	Control	49	7.4±4.9	8.6±5.1			
	Intervention	66	9.9±6.6	8.9±6.5	−1.7 (−3.1 to −0.3)	**0.02**	−0.29 (−0.52 to −0.05)
MBI personal accomplishment	Control	49	34.2±6.3	34.1±5.9			
	Intervention	66	33.7±6.5	34.8±6.3	1.0 (−0.8 to 2.8)	0.28	
PSS10	Control	49	22.1±5.5	21.4±5.7			
	Intervention	66	22.5±6.5	18.5±6.0	−3.2 (−4.9 to −1.6)	**<0.001**	−0.53 (−0.81 to −0.26)
PHQ-8	Control	48	8.2±4.0	8.2±4.2			
	Intervention	63	8.3±5.3	6.1±4.6	−2.1 (−3.5 to −0.7)	**0.003**	−0.43 (−0.73 to −0.14)
GAD-7	Control	48	7.0±4.0	7.3±4.6			
	Intervention	63	7.6±5.0	5.1±4.0	−2.4 (−3.7 to −1.1)	**<0.001**	−0.53 (−0.81 to −0.25)

GAD-7, generalised anxiety disorder seven-item scale; MBI, Maslach Burnout Inventory; PHQ-8, eight-item patient health questionnaire depression scale; PSS10, perceived stress scale.

At the end of the intervention, participants in the intervention group had significantly lower scores of emotional exhaustion as the primary outcome as well as of depersonalisation, perceived stress, depression and anxiety. Differences in personal accomplishment did not reach statistical significance. A sensitivity analysis for the primary outcome adjusting for gender and ethnicity did not significantly alter the effect size.

### Harms

No adverse events were reported.

### Additional analyses

In the intervention group, 3-month follow-up data were collected from 60 (79%) of 76 participants. The results are shown in table 3.

**Table 3:** Outcomes at the end of the intervention, 3 months later and mean change adjusted for cluster effects in the intervention group

Outcomes	N	End int. Mean±SD	Follow-up Mean±SD	Change Mean (95% CI)
MBI emotional exhaustion	60	26.8±9.7	25.5±10.3	−1.2 (−3.7 to 1.2)
MBI depersonalisation	60	8.8±6.2	7.9±6.3	−0.9 (−2.0 to 0.3)
MBI personal acc.	60	34.9±6.2	33.9±7.7	−1.0 (−2.5 to 0.5)
PSS10	60	18.0±5.3	17.5±6.6	−0.5 (−2.0 to 1.0)
PHQ-8	59	5.7±4.3	5.8±4.6	0.1 (−0.8 to 1.0)
GAD-7	59	4.9±3.9	5.1±3.7	0.2 (−0.7 to 1.2)

GAD-7, generalised anxiety disorder seven-item scale; MBI, Maslach Burnout Inventory; PHQ-8, eight-item patient health questionnaire depression scale; PSS10, perceived stress scale.

There were no substantial changes in outcomes in the intervention group during the 3-month follow-up. Improvements achieved during art therapy were largely sustained.

### Feedback questionnaire

Feedback forms were returned by 52 participants from the intervention group and 34 from the control group who received art therapy after completing their waiting period. All respondents indicated that they would recommend this type of intervention to colleagues, and 98% (n=84) found the sessions ‘helpful’ or ‘very helpful’. Two found them ‘neither helpful nor unhelpful’ and nobody rated them as ‘unhelpful’ or ‘very unhelpful’; 97% (n=83) of respondents thought that group reflective discussion was helped by the art making, while three were not sure. Most respondents (n=76) noticed a positive change in how they felt in themselves which they attributed to their participation in art therapy.

## Discussion

### Statement of principle findings

In this multisite randomised waitlist-controlled trial, group art therapy was effective in reducing burnout and mental distress in HCPs in acute hospitals. Significant improvements were found in the primary outcome of emotional exhaustion as a key dimension of burnout as well as in four out of five secondary outcomes: depersonalisation, perceived stress, depression and anxiety. The intervention was safe and acceptable, as indicated by no adverse events and by participant feedback.

### Strengths and weaknesses

This is the first full RCT to test group art therapy for HCP burnout. The study was adequately powered, and we recruited the target sample size. The follow-up rate for the primary outcome time point was 89%, within the predicted range for attrition in the study plan. No imputation was required due to minimal missing data, and only complete data samples were used in the ITT analysis. The use of medium severity clinical cut-offs at baseline allowed for clear clinical outcomes, often missing from interventional studies with HCPs.^42^ Involvement of a PPIE advisory group ensured good acceptability of the intervention. Quality assurance and replicability were supported by an intervention manual, regular supervision for the art therapists and adherence monitoring. Adherence rates of over 90%, reported by therapists and observers, indicate fidelity and deliverability of the intervention. The pragmatic nature of complex intervention delivery by NHS art therapists embedded in clinical teams assured high external validity. The study’s recruitment strategy was informed by the National Institute for Health and Care Research (NIHR) ‘Include’ framework for improving representation of underserved groups in clinical research.^30^ Finally, 43% of the trial cohort were of non-white ethnicity, therefore increasing generalisability of results to ethnic groups under-represented in mental health research.^43^

However, the study also has limitations: first, due to the waitlist design, the 3-month follow-ups were administered to the intervention group only and follow-up data had no comparator. Second, the study statistician generated the randomisation sequence and, third, the sample size was too small to identify significant changes in professional subgroups. As this is the first RCT of its kind, there is no established effect size for group art therapy for HCP burnout. We, therefore, used cognitive behavioural therapy for a related disorder (anxiety) in a psychiatric population^44^ to design our study. We are just beginning to understand this area and anticipate that, in future studies, the validity of certain effect sizes and an understanding of how these translate to clinical effectiveness will be established.

### Comparison with other studies

Interventions to treat HCP burnout have been reported to have small or negligible effects,^20^,^22^ and studies of various interventions, including discussion groups^20^ and cognitive therapy,^45^ have failed to demonstrate relevant change in burnout compared with no intervention. Other studies, such as those assessing psychoeducation,^46^ mindfulness^47^ and organisation-based interventions, such as adjusted work scheduling,^22 48^ have found significant but modest changes in emotional exhaustion. Systematic reviews report data inconsistency and high risk of bias, limiting the reliability of pooled results.^19^,^21^ A meta-analysis of randomised trials testing interventions to address physician burnout found a −1.1-point mean difference between groups (95% CI 2.14 to 0.09) in emotional exhaustion, which was deemed numerically significant but unlikely to be clinically meaningful.^22^ In this trial, art therapy was associated with an adjusted −4.8-point difference (95% CI −7.3 to −2.4) in emotional exhaustion, as well as larger differences in depersonalisation than typically seen.^22^

Despite the context of disruptive industrial action (by junior and consultant doctors, nurses and transport providers) and unprecedented work pressure, which impacted participants during the study period,^49^ the medium effect sizes attributable to the intervention were notably larger than effects typically observed in other burnout interventions aimed at HCPs.^20^,^22^ As also seen in pooled mean results from previous studies,^22^ we found no significant differences between groups for personal accomplishment. The larger effect seen for emotional exhaustion than the other two burnout dimensions in ours and previous studies is likely because emotional exhaustion is often the first and most pronounced symptom of burnout, and its impact on daily functioning and well-being can lead to a greater measurable effect.^10^

Two previous pilot RCTs have assessed group art therapy methods for HCP burnout. The first, also using a waitlist control design, investigated a 6-week multimodal intervention of Mindful-Compassion Art-based Therapy (MCAT) for reducing risk of burnout in home hospice workers in Singapore. The findings indicated a positive change in emotional exhaustion, also sustained at 3-month follow-up as in our study.^50^ The second study explored the outcomes of creative arts therapy (CATS) versus no treatment in an acute hospital setting in the USA.^51^ They also reported a positive change in emotional exhaustion. However, participants were spread between four separate CATS modalities, that is, visual arts, music, dance and writing. Our trial, which focused purely on art therapy, had a more ethnically and professionally diverse sample, which included higher participation from doctors than these pilot trials.

### Meaning of the study

The intervention offered six sessions of group art therapy of which a median of four was attended. This brief intervention significantly reduced risk of burnout and levels of mental distress in a heterogeneous sample of HCPs. This is notable, as significant change across both occupational burnout and mental health outcomes is rarely seen in HCP interventions.^20 45^ There are several aspects that may have contributed to this positive effect. First, HCPs are likely to have been well motivated. HCPs from different specialties and backgrounds came forward and wanted to participate in this voluntary intervention, even though participation was outside working hours and not associated with any formal advantage or reward. Mandatory well-being programmes can have negative associations for HCPs,^52^ and the voluntary nature of participation in this trial may have contributed to a higher motivation. Second, the level of motivation to participate was largely maintained during therapy, possibly because of the nature of the specified form of art therapy. Attendance was good despite challenges, such as industrial action, and there was anecdotal evidence that some HCPs came to the hospitals before their night shifts and even during days off to participate in the groups. Third, the indirect, non-verbal approach of art therapy may have made it easier for many HCPs to engage in the process.^53^ Fourth, the specified model of group art therapy that incorporates mindfulness and psychoeducation with embodied art-based processes is likely to have offered a range of opportunities for stress reduction, gaining new perspectives, emotional expression and emotional processing, previously ascertained perceived effects of art therapy for HCPs.^23^ Finally, findings from qualitative analysis of perceived effects, which ran alongside the trial (to be reported separately), strongly suggest the intervention acted on both individual and systems levels.

Burnout in HCPs is prevalent and appears to be increasing,^8 54^ putting pressure on healthcare organisations to introduce programmes for reducing burnout. Systematic reviews recommend a mixed package of individual- and organisation-directed approaches for synergistic effectiveness to address root causes and for meaningful change to happen.^55 56^ However, empirical evidence to guide the decisions about what programmes to offer to staff is widely lacking. The findings of this trial provide evidence for the effectiveness of an acceptable, feasible, safe and reproducible intervention for HCPs in acute hospital settings. The therapy was tested in a pragmatic trial in the real clinical world and has a flexible and adaptable format: it is provided in groups; HCPs from all specialties and professional backgrounds can participate and it consists of only six sessions. One may conclude that such programmes should be a part of organisational and national strategies to address healthcare workforce burnout. Further trials with different and larger samples and in different countries, areas and settings will certainly be helpful. They may identify differentiated effects in subgroups and test whether modified approaches may be even more effective. However, for the time being, such trials have not been conducted and decisions need to be based on the evidence that is available now. Given the current crisis of burnout in HCPs and the lack of other similarly feasible and effective interventions, it is difficult to justify holding back with rolling out the intervention across hospitals now wherever possible.

### Unanswered questions and further research

The intervention is delivered in groups with only one therapist present, and the groups can happen outside working hours. Thus, the costs for the employer organisation are very limited and certainly justified, given the positive outcomes. However, the study did not include an economic evaluation, and the costs of implementation should be formally established in further research to guide the decision of provider organisation for the roll-out of the intervention. Some practical challenges remain. For example, in some areas, there may not be enough qualified art therapists, and even qualified art therapists would require some training and supervision in the specified form of art therapy used in this trial. Further investigation into how art therapy-based interventions for staff support can become a part of routine organisation of a hospital is required. Future research should examine implementation. Another area for further exploration is dose. In this study, effects were seen following a median attendance of four sessions, but it is possible that a longer or more intensive version of the therapy could be associated with a stronger effect. Finally, further research into interventions acting on the personal accomplishment dimension of burnout is recommended.

It needs to be emphasised that the intervention tested in this study addresses burnout and distress that has already developed and is prevalent in HCPs. However, healthcare organisations should implement such interventions that does not change the need to address the root causes of burnout of HCPs through changes within organisations, healthcare systems and possibly even the wider society.

## Supplementary material

10.1136/bmjph-2024-002251 online supplemental file 1

10.1136/bmjph-2024-002251 online supplemental file 2

10.1136/bmjph-2024-002251 online supplemental file 3

10.1136/bmjph-2024-002251 online supplemental file 4

## Data Availability

Data are available on reasonable request.
