# Supplementary material for: Art therapy to reduce burnout and mental distress in healthcare professionals in acute hospitals: a randomised controlled trial
Source: BMJ Public Health. 2025 Aug 3;3(2):e002251. doi: 10.1136/bmjph-2024-002251 (PMC12320087; doi:10.1136/bmjph-2024-002251)
Supplement: online supplemental file 1 [file bmjph-3-2-s001.docx]

Supplement 1 - Study procedures

|  | Visits |  |  |  |  |  |  |  |  |  |
| --- | --- | --- | --- | --- | --- | --- | --- | --- | --- | --- |
| Procedures | Screening | Baseline | Treatment |  |  |  |  |  | 1 week post intervention | 12 weeks post intervention |
|  |  |  | 1 | 2 | 3 | 4 | 5 | 6 |  |  |
| Initial contact expressing interest in study | X |  |  |  |  |  |  |  |  |  |
| Screening email | X |  |  |  |  |  |  |  |  |  |
| Informed Consent email/ phone | X |  |  |  |  |  |  |  |  |  |
| Demographics |  | X |  |  |  |  |  |  |  |  |
| Randomisation |  | X |  |  |  |  |  |  |  |  |
| MBI-HSS |  | X |  |  |  |  |  |  | X | X |
| GAD-7 |  | X |  |  |  |  |  |  | X | X |
| PHQ-8 |  | X |  |  |  |  |  |  | X | X |
| PSS-10 |  | X |  |  |  |  |  |  | X | X |
| Group art therapy (intervention arm) |  |  | X | x | X | x | x | x |  |  |
| Feedback questionnaire |  |  |  |  |  |  |  |  | X |  |
