# Supplementary material for: Art therapy to reduce burnout and mental distress in healthcare professionals in acute hospitals: a randomised controlled trial
Source: BMJ Public Health. 2025 Aug 3;3(2):e002251. doi: 10.1136/bmjph-2024-002251 (PMC12320087; doi:10.1136/bmjph-2024-002251)
Supplement: online supplemental file 2 [file bmjph-3-2-s002.docx]

| TiDier item | Description |
| --- | --- |
| 1. Name of intervention | Group art therapy for healthcare professionals |
| 1. Why this intervention? | A systematic review provides compelling evidence that group art therapy reduces burnout and mental distress in healthcare workers (Tjasink et al 2023). A version of the intervention, piloted with oncology and palliative care doctors was found to be effective for reduction of burnout, acceptable to participants and feasible for delivery within an acute hospital setting (Tjasink and Soosaipillai 2019). |
| 1. What materials were used? | Range of wet and dry art materials, e.g. acrylic paints, Indian inks, water-soluble coloured pencils, chalk pastels, oil pastels, graphite pencils, charcoal.  Modelling material, e.g. air-drying clay, plasticine.  Non-traditional materials, e.g. rubbing alcohol, salt, wax candles  Paper and card in white, black and a range of colours and sizes  Craft supplies, e.g. scissors, Sellotape, water-based glue, coloured tissue paper, string, ribbon.  Other equipment for e.g. blue tac, table covers, plastic aprons |
| 1. What procedures were used? | Group art therapy with exploratory, expressive art making, adapted from an art therapy programme for oncology and palliative care doctors (Tjasink et al) and informed by biopsychosocial therapeutic models such as Compassion Focussed Therapy (Gilbert, 2014) and trauma – informed arts therapy (Labban, 2017). |
|  | 4.1 Art psychotherapist providers induction and training, involving seminars and shadowing |
|  | 4.2 Participants sent an email in advance of their start date with two documents, the first titled “What to expect: CHArt art therapy group programme” and the second “CHArt code of conduct” which included notes on psychological safety. This email included a reminder of their session dates to encourage attendance. |
|  | General group structure.Groups are structured but not inflexible. Each session follows a similar format including: 1. Socialisation – arriving, greeting each other, making tea. 2. Introduction to the workshop theme, art materials and processes. 3. Art – based warm up or grounding exercise. 4. Individual or collaborative art making. 5. Sharing and viewing art products and processes. 6. Reflective group discussion. 7. Tidy up, storing artwork. |
|  | 4.4 Session 1: Introductions, group code of conduct and psychological safety, introduction to art therapy. Speed drawing and mandalas. Session 2: Exploring natural objects and experimental art making. Session 3: Working with clay – individual grounding exercise followed by collaborative art making. Session 4: Transforming images (problem solving) – collaborative and group art making. Session 5: Create, destroy, transform – process based, trauma – informed exercise. Session 6: Reflection and consolidation. |
|  | 4.5 Clinical supervision for art psychotherapist providers. |
| 1. Who provided it? | HCPC registered NHS art psychotherapists |
| 1. How was it provided? (e.g. group or individual) | In person, group format, up to 10 participants per group |
| 1. Where did it take place? | 5 seminar rooms within 4 acute hospital settings. Rooms varied but all could seat up to 10 participants around a large table, had access to a nearby sink and adequate space to lay out art materials. Rooms were away from wards and free from interruption for the duration of the session. |
| 1. When and How Much? (Number, frequency, duration of sessions) | 6 sessions of 90 minutes each delivered weekly for a 6-week period. (Where bank holidays, room or therapist availability intervened, sessions spanned 7 – 8 weeks). |
| 1. Tailoring | The sequence of themes was adjusted in one group due to low attendance on a day scheduled for collaborative group art making. |
| 1. Modifications | Participants unable to attend regularly were supported to stay in contact with the lead art psychotherapist and to return when they could. This meant some participants attended only once or twice. Linked to this, the final session was adapted to include an option to catch up on a previous missed session activity. |
| 1. How well was it delivered? (Planned fidelity strategies and adherence) | A pre-designed adherence checklist was completed by providers every session  An observer-rated adherence checklist was completed by independent clinician rater. |
| 1. How well was it delivered? (Actual) | Mean adherence of 98.3% (therapist rated) with a confidence rating of 91.7%. |
