# Supplementary material for: Art therapy to reduce burnout and mental distress in healthcare professionals in acute hospitals: a randomised controlled trial
Source: BMJ Public Health. 2025 Aug 3;3(2):e002251. doi: 10.1136/bmjph-2024-002251 (PMC12320087; doi:10.1136/bmjph-2024-002251)

Supplement 3 – Example Artwork

Example artwork from Session 2: “Exploring natural objects and experimental art making”


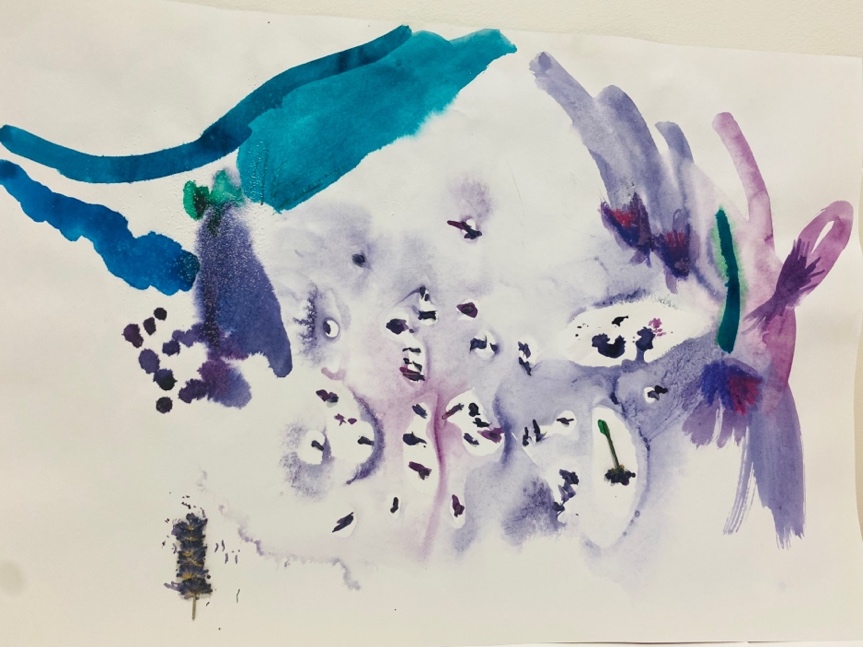

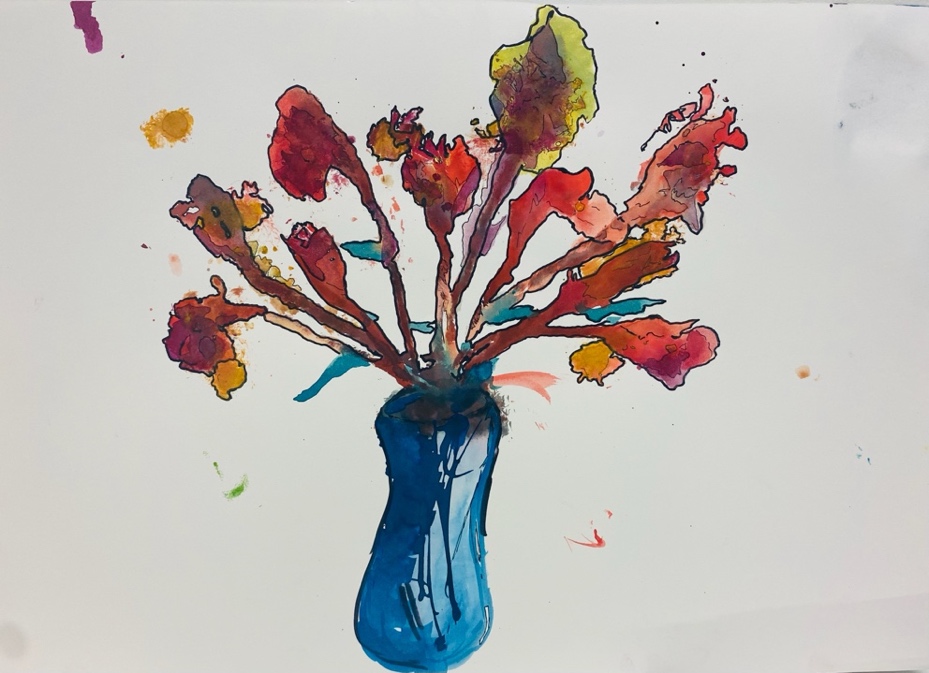


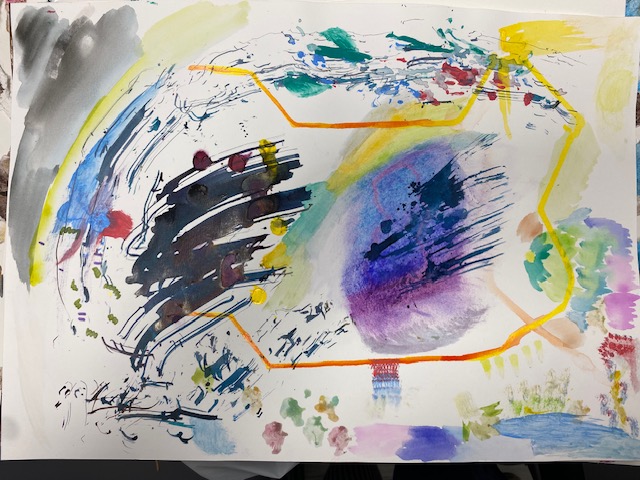

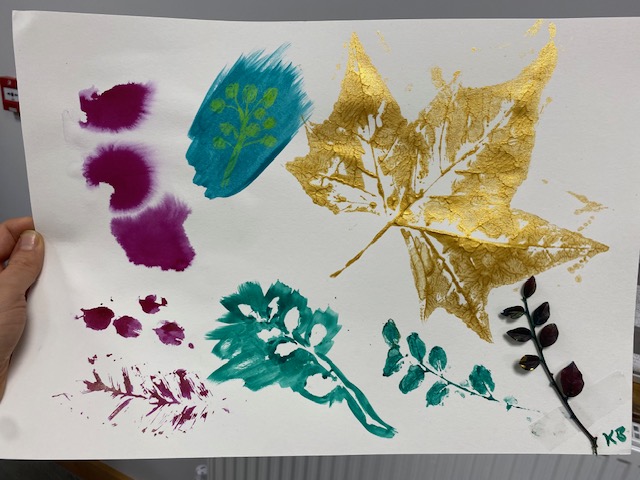


Example artwork from Session 4: “Transforming images (collaborative problem solving)”


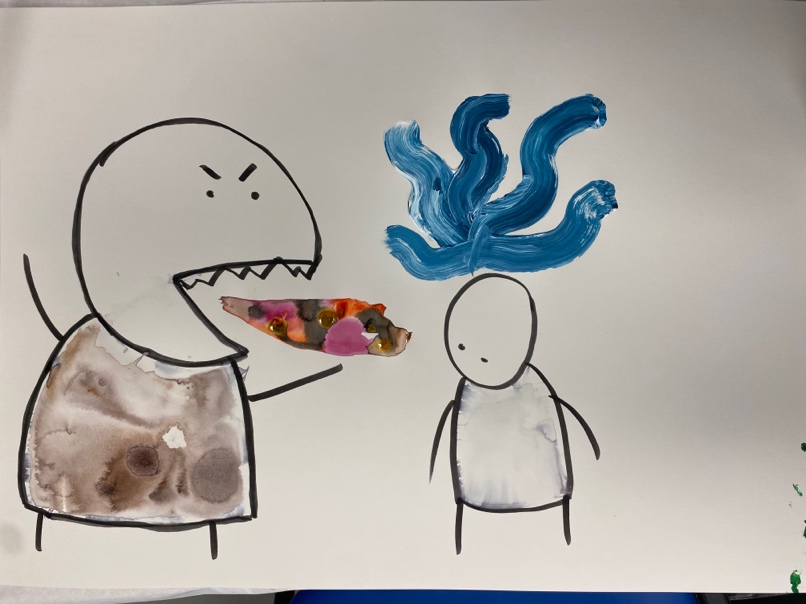

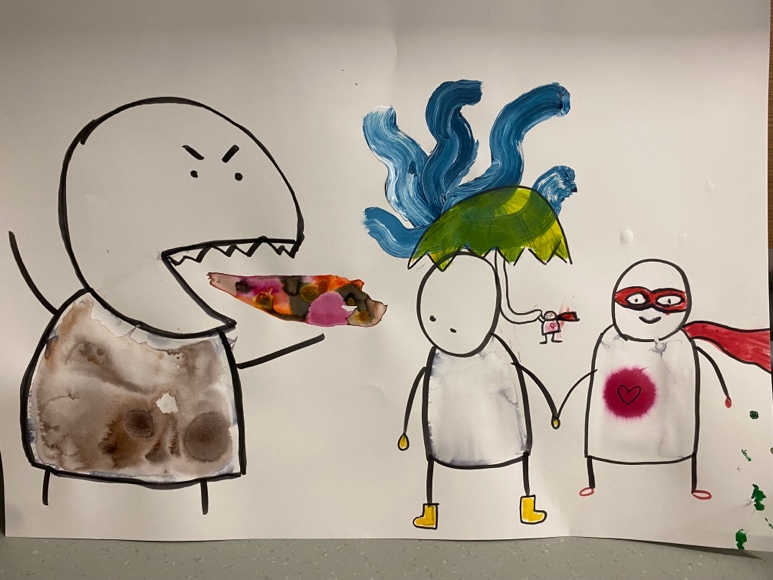


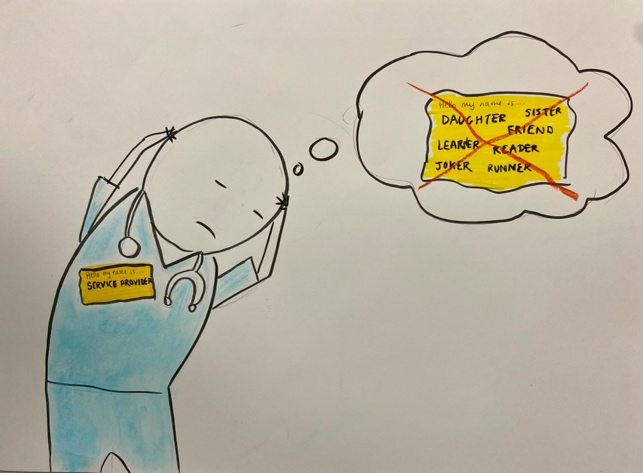

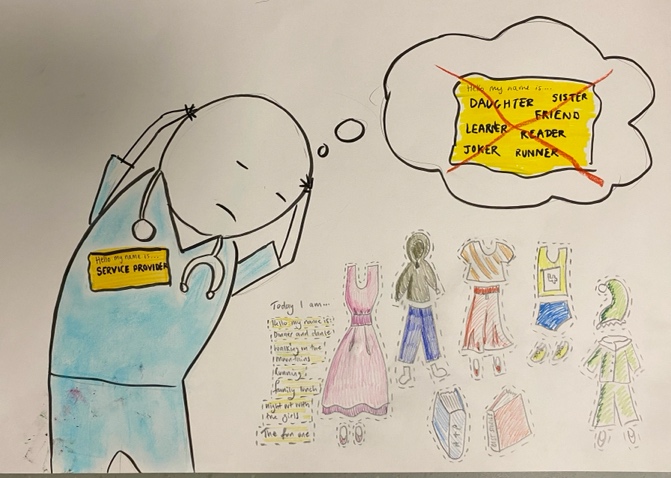


Example artwork from Session 5: “Create, destroy, transform”


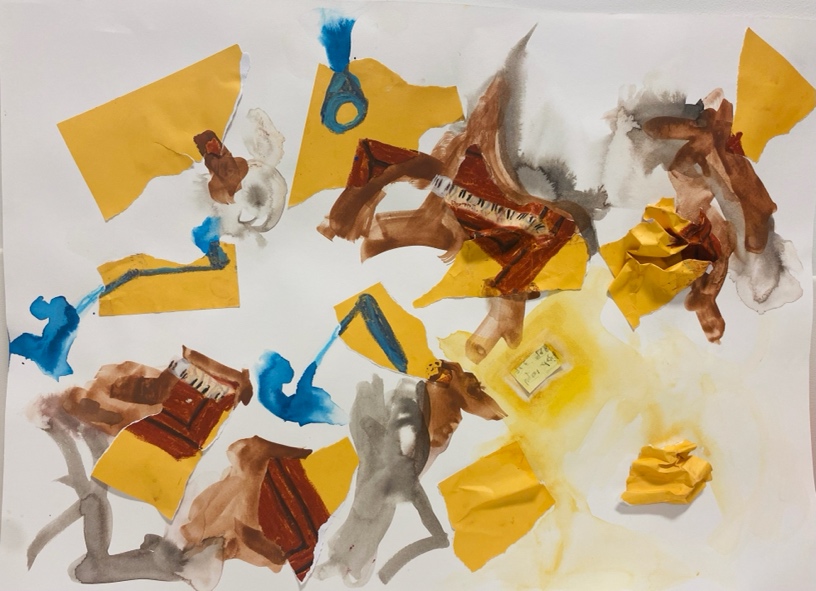

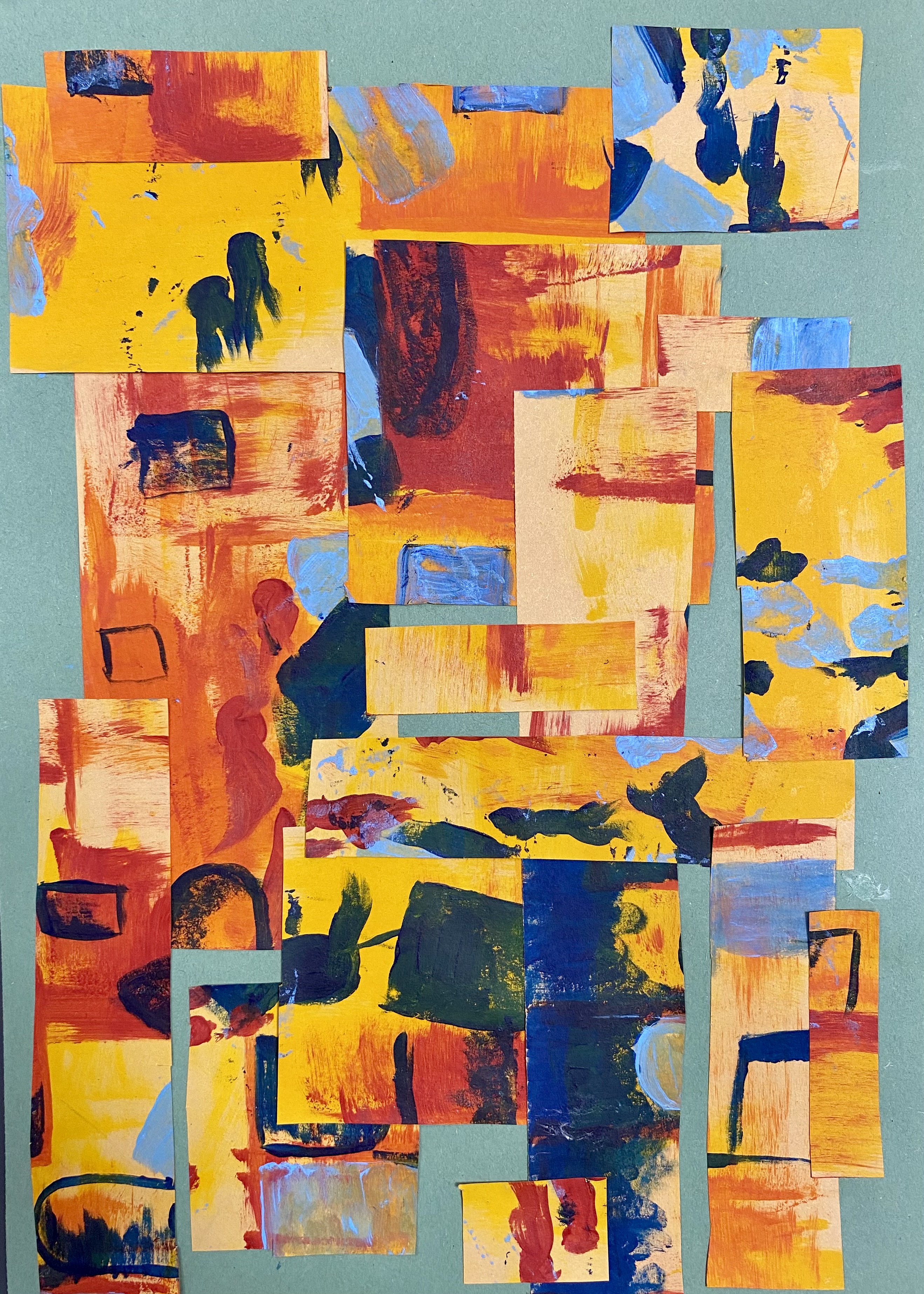

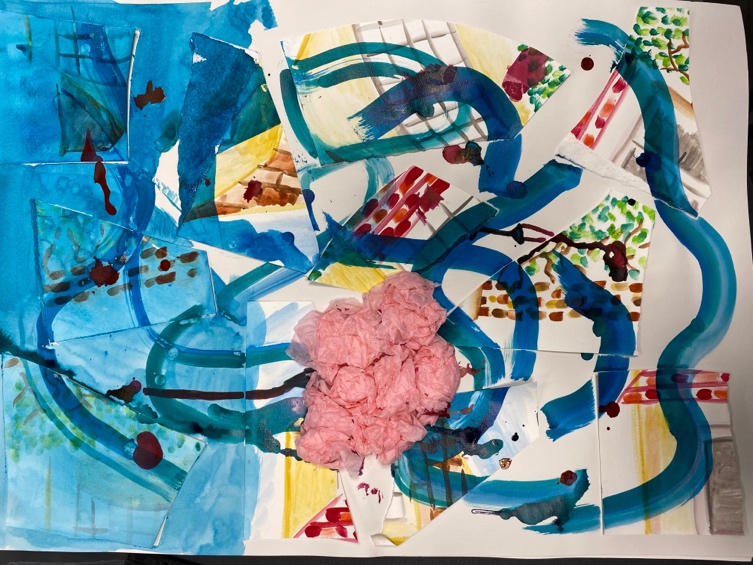

Supplement: online supplemental file 3 [file bmjph-3-2-s003.docx]
