# Supplementary material for: Art therapy to reduce burnout and mental distress in healthcare professionals in acute hospitals: a randomised controlled trial
Source: BMJ Public Health. 2025 Aug 3;3(2):e002251. doi: 10.1136/bmjph-2024-002251 (PMC12320087; doi:10.1136/bmjph-2024-002251)
Supplement: online supplemental file 4 [file bmjph-3-2-s004.docx]

**CHArt Study Therapist self-adherence form**

**DATE ___ / ___ / ________ SESSION NUMBER: _____**

**THERAPIST: ____________ HOSPITAL SITE:________________**

*Please rate if the following happened in your session today and how confident you were, using the scale below:*

**Confidence rating scale:**

| Not applicable | Very unconfident | Somewhat unconfident | Neutral (neither confident nor unconfident) | Somewhat confident | Very confident |
| --- | --- | --- | --- | --- | --- |
| 999 | 1 | 2 | 3 | 4 | 5 |

|  | Did this happen?  *Write Yes/No* | How confident were you?  *Use scale above* | If this did not happen or confidence is 3 or less, place a tick here to take to supervision. *Tick* |
| --- | --- | --- | --- |
| 1. Engagement, interest and warmth |  |  |  |
| 2. Transparency, examples and explanations |  |  |  |
| 3. Use of psycho-education (informed by biopsychosocial / body-mind model/CFT/ACT/ evolutionary psychology) |  |  |  |
| 4. Exploration, curiosity, encouraging new perspectives |  |  |  |
| 5. Compassionate, non-judgemental approach |  |  |  |
| 6. Encouragement of group cohesion and collegiate connection |  |  |  |
| 7. Normalising a range of feelings |  |  |  |
| 8. Group structure as outlined in the manual |  |  |  |
| 9. Group activity as outlined in the manual |  |  |  |
| 10. Encouraging engagement and exploration of art materials and processes |  |  |  |
| 11. Modelling use and potentials of art materials and processes |  |  |  |
| 12. Focus on arts-based, embodied, emotional experiences |  |  |  |
| 13. Encouragement of “bottom – up” processing through art making, the senses and feelings |  |  |  |
| 14. Paying attention to the here and now / being in the moment |  |  |  |
